# Development of a new intraocular lens power calculation method based on lens position estimated with optical coherence tomography

**DOI:** 10.1038/s41598-020-63546-y

**Published:** 2020-04-16

**Authors:** Tsukasa Satou, Kimiya Shimizu, Shuntaro Tsunehiro, Akihito Igarashi, Sayaka Kato, Manabu Koshimizu, Takahiro Niida

**Affiliations:** 1 Department of Orthoptics and Visual Sciences, School of Health Sciences, International University of Health and Welfare, Tochigi, Japan; 2 Sanno Hospital, Tokyo, Japan; 3 International University of Health and Welfare, Tochigi, Japan

**Keywords:** Outcomes research, Imaging and sensing

## Abstract

A new method is developed and validated for intraocular lens (IOL) power calculation based on paraxial ray tracing of the postoperative IOL positions, which are obtained with the use of anterior segment optical coherence tomography. Of the 474 eyes studied, 137 and 337 were grouped into training and validation sets, respectively. The positions of the implanted IOLs of the training datasets were characterized with multiple linear regression analyses one month after the operations. A new regression formula was developed to predict the postoperative anterior chamber depth with the use of the stepwise analysis results. In the validation dataset, postoperative refractive values were calculated according to the paraxial ray tracing of the cornea and lens based on the assumption of finite structural thicknesses with separate surface curvatures. The predicted refraction error was calculated as the difference of the expected postoperative refraction from the spherical-equivalent objective refraction values. The percentage error (within ±0.50 diopters) of the new formula was 84.3%. This was not significantly correlated to the axial length or keratometry. The developed formula yielded excellent postoperative refraction predictions and could be applicable to eyes with abnormal proportions, such as steep or flat corneal curvatures and short and long axial lengths.

## Introduction

It is common knowledge that cataract surgery includes elements of refractive and presbyopic surgery in addition to the removal of the diseased tissue^1–5^. The implantation of an intraocular lens (IOL) in conjunction with the appropriate power calculation improves patient satisfaction levels and leads to successful outcomes.

The most important aspect associated with the reduction of the refractive prediction error is the accurate prediction of postoperative optical biometry based on preoperative data. Norrby *et al*.^6^ demonstrated that preoperative estimation of the postoperative IOL position contributed to IOL power prediction errors. The prediction of the postoperative IOL position was associated with the largest contribution to the error in compound IOL power prediction. The postoperative anterior chamber depth (ACD) is accounted for in the IOL power calculation formula (i.e., effective lens position: ELP) and is formula-dependent. However, it does not reflect the true postoperative ACD in the anatomical sense. In particular, this tendency is noticeable in the third-generation IOL power calculation formulas (Hoffer Q^7^, Holladay 1^8^, and SRK/T^9^). In the Holladay 1 and SRK/T formulas, the ELP was calculated to be deeper or shallower in the respective cases of steep or flat corneal curvature radii based on estimates which relied on the Pythagorean theorem with corneal curvature radius data^10^. This method was developed by Fyodorov^11^.

Ray tracing is a validated and useful approach employed for theoretical IOL power calculations^12–17^. Olsen^12^ reported a paraxial thick lens formula (paraxial ray tracing) based on the theory of Gaussian optics in 1987. Additionally, the exact ray tracing considered in conjunction with the pupil size and higher-order aberrations can yield a physically correct interpretation^15^. In any case, the true postoperative ACD is necessary to yield a successful performance in the case of the ray-tracing method. IOL power values can be calculated with greater accuracy using ray tracing programs that incorporate accurate predictions of the postoperative ACD. Anterior segment optical coherence tomography (AS–OCT) achieves a highly accurate prediction of the IOL position. We previously reported the relationship between the crystalline lens parameter and the IOL position^18^. Goto *et al*.^19^ developed a formula to predict postoperative ACD, including the angle-to-angle (ATA) depth obtained by AS–OCT. Enriquez *et al*.^20^ presented a method to accurately estimate and quantify the entire shape of the crystalline lens from OCT data, and reported that the preoperative anterior segment parameters were exceptionally valuable for estimating the IOL position. Yoo *et al*.^17^ reported improvements in refractive outcomes by the ray-tracing method using the equatorial plane position of the lens.

Paraxial ray tracing repeats simple calculations without special software or complexity. The predictive accuracy of IOL power calculations with paraxial ray tracing including the IOL positions predicted by the AS–OCT has not been clarified.

Correspondingly, the aims of the present study are a) the development of a new formula based on paraxial ray tracing for IOL power calculations that utilize the postoperative IOL positions obtained using AS–OCT, and b) the validation of the accuracy of the developed formula.

## Results

In total, 474 eyes of 474 Japanese patients were enrolled in this study, and were classified into a training set (n = 137) and a validation set (n = 337) (See Supplementary Materials for minimum data set). Demographic data are shown in Table 1. The axial length (AL), keratometry (K values), ACD, and crystalline lens thickness (LT) measured by the IOLMaster 700 (ILM700) in the training set were not significantly different compared to those in the validation set.

**Table 1:** Demographic data in training and validation set.

Parameter	Training set (137 eyes)			Validation set (337 eyes)			P value
	Mean ± SD	Minimum	Maximum	Mean ± SD	Minimum	Maximum	
Age (years-old)	70 ± 11	26	90	70 ± 10	33	104	0.664
Females, n (%)	90 (66)			188 (56)			0.047
Axial length (mm)	24.42 ± 1.52	21.72	28.47	24.55 ± 1.77	21.42	30.78	0.437
Keratometry (D)	43.97 ± 1.38	40.35	48.39	43.83 ± 1.51	38.94	48.12	0.351
Central corneal thickness (μm)	551 ± 32	464	641	560 ± 37	452	648	0.006
Anterior camber depth (mm)	3.14 ± 0.39	2.19	4.26	3.16 ± 0.38	1.96	4.34	0.638
Crystalline lens thickness (mm)	4.53 ± 0.42	3.64	5.49	4.54 ± 0.38	3.38	6.15	0.886

SD: standard deviation.

Anterior camber depth is defined as axial measurement from corneal epithelium to anterior lens.

### Use of single and multiple linear regression analyses for the development of prediction formulas of the anterior intraocular lens surface position in the training set

In the training set, the mean values measured by CASIA2 were: corneal posterior curvature radius (PCR): 6.42 ± 0.25 mm, central corneal thickness (CT): 0.54 ± 0.04 mm, ATA depth: 3.27 ± 0.20, ATA width: 11.48 ± 0.42 mm, anterior surface depth (ASD): 2.72 ± 0.38 mm, equatorial surface depth (ESD): 4.18 ± 0.35 mm, and posterior surface depth (PSD): 7.26 ± 0.31 mm. The mean corneal anterior curvature radius (ACR) value measured by the ILM700 was 7.68 ± 0.24 mm. In addition, the mean correct AL value calculated by the formula described in the Methods section was 24.57 ± 1.47 mm. The mean anterior IOL surface position measured postoperatively (at one month) was 4.01 ± 0.30 mm.

The single and multiple linear regression analysis outcomes are listed in Table 2. In the single linear regression analysis, the anterior IOL surface position yielded significant correlations with seven preoperative variables, including the PCR, ATA depth, ATA width, ASD, ESD, PSD, and the correct AL value. In the multiple linear regression analysis, the combination of the ATA depth, PSD, ACR, and the correct AL value yielded the highest correlation with the anterior IOL surface position. The following formula for the prediction of the anterior IOL surface position was developed based on the results of the stepwise analysis:

1 $$Predictive\,IOL\,position=0.513\times ATA\,depth+0.345\times PSD+0.098\times predicted\,true\,AL-0.255\times ACR\mbox{--}0.603$$

**Table 2:** Single and multiple linear regression analysis for prediction of anterior intraocular lens surface position.

Training set (137 eyes)				
Variable	Correlation coefficients by single linear regression analysis	P value	Partial regression coefficients of selected variable by multiple linear regression analysis (stepwise)	P value
**Variable obtained by CASIA2**				
Corneal posterior curvature radius	0.181	0.034		
Central corneal thickness	−0.052	0.543		
Angle to angle depth	0.683	<0.001	0.354	<0.001
Angle to angle wide	0.439	<0.001		
Anterior surface depth of crystalline lens	0.663	<0.001		
Equatorial surface depth of crystalline lens	0.641	<0.001		
Posterior surface depth of crystalline lens	0.721	<0.001	0.358	<0.001
**Variable obtained by IOL Master 700**				
Corneal anterior curvature radius	0.081	0.348	−0.209	<0.001
Correct AL	0.662	<0.001	0.485	<0.001

When this formula was used for the training set, the anterior IOL surface position was predicted with a total determination coefficient (R^2^) of 0.804, while the intraclass correlation coefficient (ICC) was 0.892.

### Development of a new formula with paraxial ray tracing

The refractive power of each refractive surface was calculated as follows,

2 $${D}_{1}=\frac{{n}_{1}-{n}_{2}}{{r}_{1}}\,{D}_{2}=\frac{{n}_{2}-{n}_{3}}{{r}_{2}}\,{D}_{3}=\frac{{n}_{3}-{n}_{4}}{{r}_{3}}\,{D}_{4}=\frac{{n}_{4}-{n}_{5}}{{r}_{4}}$$

where *D*_1_ = corneal anterior refractive power, *D*_2_ = corneal posterior refractive power, *D*_3_ = anterior refractive power of IOL, *D*_4_ = posterior refractive power of IOL, *r*_1_ = ACR, *r*_2_ = PCR, *r*_3_ = anterior curvature radius of IOL at each power level, *r*_4_ = posterior curvature radius of IOL at each power, *n* = refractive index, *n*_1_ = 1.0003 (air), *n*_2_ = 1.376 (cornea), *n*_3_ = 1.336 (aqueous humor), *n*_4_ = 1.413 (IOL), and *n*_5_ = 1.336 (vitreous humor). The expected postoperative refractive values were calculated as follows:

3 $$Vergence\,at\,posterior\,surface\,of\,IOL\,({V}_{1})=\frac{{n}_{5}}{-e}$$

4 $$Vergence\,at\,anterior\,surface\,of\,IOL\,({V}_{2})=\frac{{n}_{4}}{\frac{{n}_{4}}{{V}_{1}+{D}_{4}}-d}$$

5 $$Vergence\,at\,posterior\,surface\,of\,cornea\,({V}_{3})=\frac{{n}_{3}}{\frac{{n}_{3}}{{V}_{2}+{D}_{3}}-c}$$

6 $$Vergence\,at\,anterior\,surface\,of\,cornea\,({V}_{4})=\frac{{n}_{2}}{\frac{{n}_{2}}{{V}_{3}+{D}_{2}}-b}$$

7 $$Expected\,postoperative\,refractive\,value=\frac{-{n}_{1}}{\frac{{n}_{1}}{{V}_{4}+{D}_{1}}-a}$$

where *a* = vertex distance (VD) (12 mm), *b* = CT obtained by CASIA2, *c* = predicted IOL position, *d =* IOL thickness at each power level, and *e* = vitreous length (correct AL value-*b*-*c*-*d*).

### Validation of the prediction accuracy of the new formula

The prediction accuracy of the new formula was compared with those associated with the Barrett Universal II, Haigis, and SRK/T formulas based on the validation set. Based on the optimized lens constant from the training set, the following values were used: lens factor of the Barrett Universal II formula (1.75), a0 of the Haigis formula (1.45), and A constant of the SRK/T formula (118.74). The mean IOL power was +19.32 ± 4.03 diopter (D) (range +7.00 to +26.00 D).

**Figure 1:**
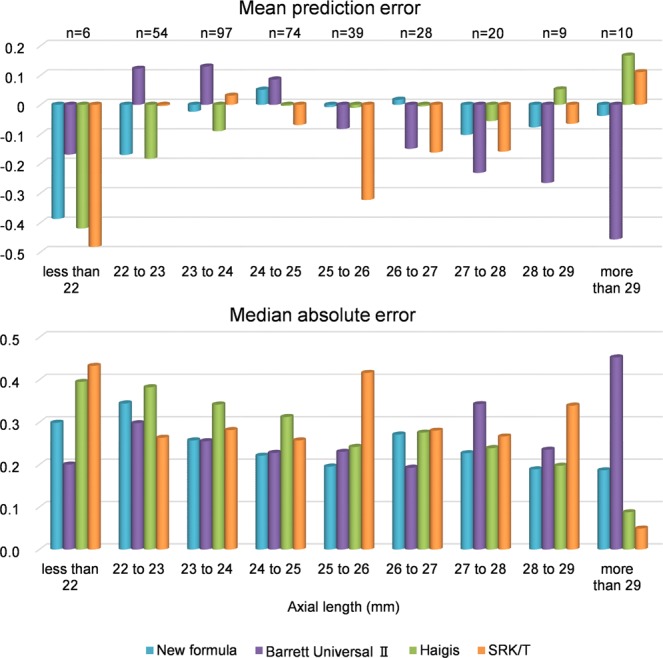
Mean prediction errors and medians absolute errors of studied axial length groups.

**Figure 2:**
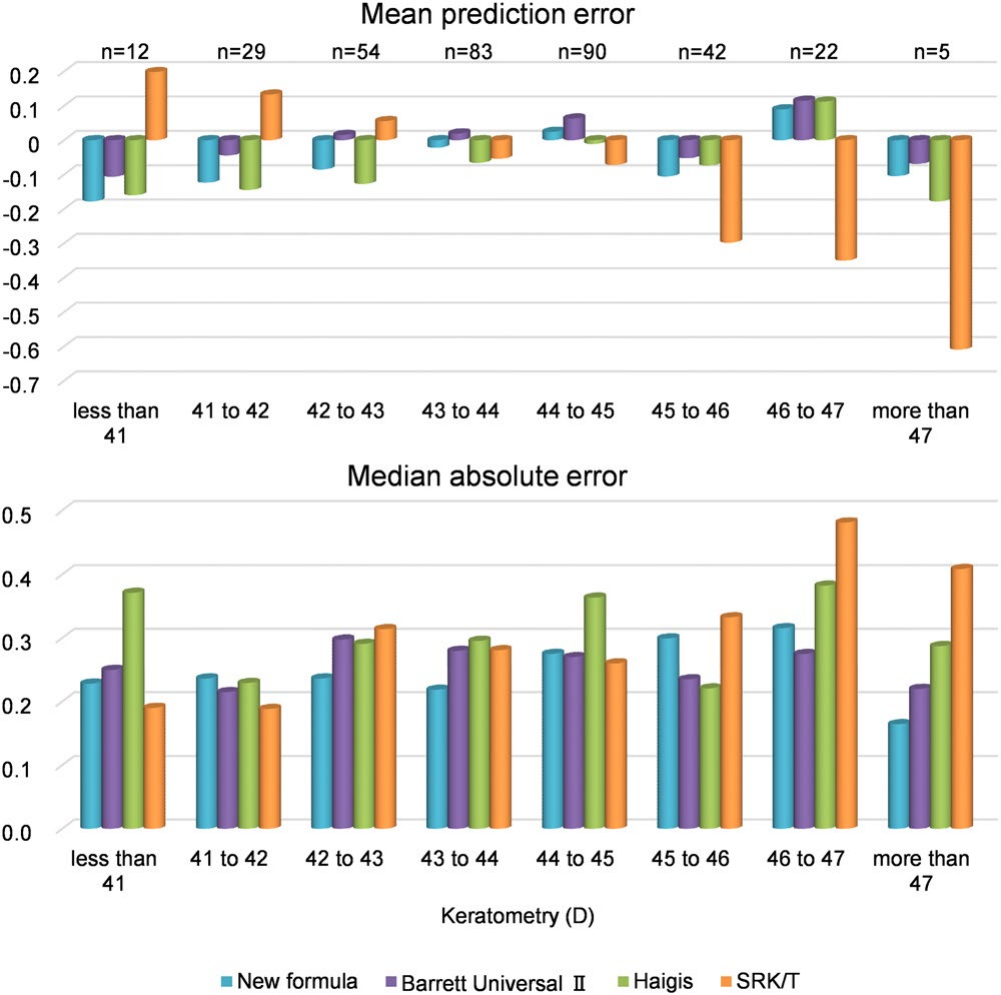
Mean prediction errors and medians absolute errors of each studied keratometry groups.

**Figure 3:**
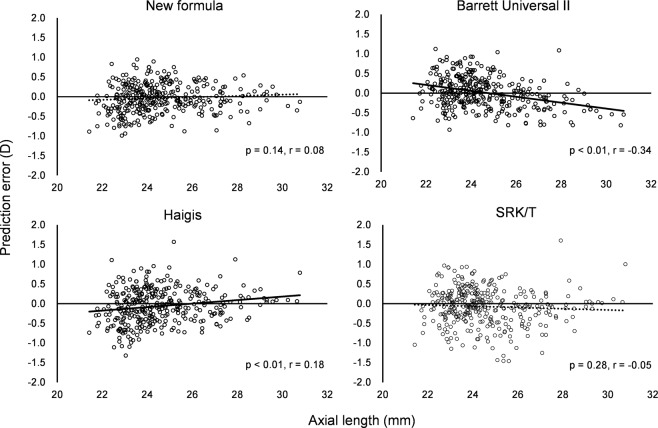
Correlations between prediction errors and axial lengths.

**Figure 4:**
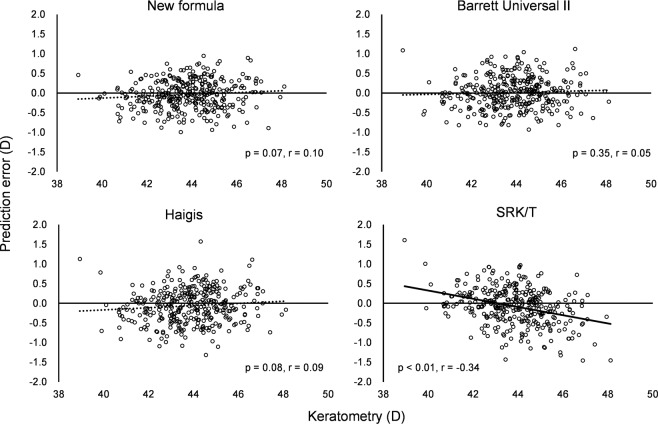
Correlations between prediction errors and keratometric measurements.

The predicted refraction errors of all formulas are listed in Table 3. The mean refraction prediction error (PE) is −0.04 ± 0.36 D in the case of the new formula, +0.02 ± 0.40 D in the case of the Barrett Universal II formula, −0.06 ± 0.44 D in the case of the Haigis formula, and −0.07 ± 0.47 D in the case of the SRK/T formula. The median absolute error (MedAE) in the new formula is 0.25 D, which is significantly different from those associated with the Haigis formula (0.30 D, *P* < 0.001) and the SRK/T formula (0.27 D, *P* = 0.009), but insignificantly different from the error associated with the Barrett Universal II formula (0.27 D, *P* = 0.361). The percentage error of the new formula (within ± 0.50 D) is 84.3%, which is significantly different from those associated with the Barrett Universal II formula (78.0%, *P* = 0.036), Haigis formula (75.1%, *P* < 0.001), and the SRK/T formula (73.9%, *P* < 0.001). The percentage error of the new formula (within ± 1.00 D) is 100%, which is significantly different from that associated with the SRK/T formula (96.1%, *P* = 0.003), but not significantly different from those associated with the Barrett Universal II formula (99.1%, *P* = 0.745) and Haigis formula (97.9%, *P* = 0.070). These errors are analyzed for biases against the AL and K (Figs. 1–4). In any AL and K groups, the PEs in the new formula are the same or better than those associated with other formulas. PE values are significantly correlated with AL values in the cases of the Barrett Universal II and Haigis formulas (*P* < 0.01, respectively), and are significantly correlated with the K value in the SRK/T formula (*P* < 0.01), whereas the PE value of the new formula is not significantly correlated with the AL (*P* = 0.142) and K values (*P* = 0.074).

**Table 3:** Predictive refraction errors of all formulas.

	New formula	Barrett universal II	Haigis	SRK/T
Mean prediction error	−0.04	0.02	−0.06	−0.07
Standard deviation	0.36	0.40	0.44	0.47
Minimum	−0.99	−0.93	−1.32	−1.46
Maximum	0.94	1.12	1.57	1.60
Mean absolute error	0.29	0.31	0.35	0.36
Median absolute error	0.25	0.27	0.30**	0.27**
**Percentage of errors within diopter range**				
±0.25D	49.9%	48.7%	42.1%	44.8%
±0.50D	84.3%	78.0%*	75.1%**	73.9%**
±0.75D	96.1%	93.2%	92.9%	87.8%*
±1.00D	100%	99.1%	97.9%	96.1%**

Median absolute error and percentage of errors within diopter range in New formula were compared with other existing formula.

*p < 0.05, **p < 0.01.

## Discussion

The new formula developed in the current study yielded excellent predictions of postoperative refraction. The developed formula was based on thick lens calculations that considered the finite thicknesses and separate surface curvatures of the cornea and lens. When paraxial ray tracing was used in the IOL power calculation, we had to measure and use the physical distances. In the present study, the AL value measured by the ILM700 was converted to the optical path lengths (OPL) with the use of the average refractive index (1.3549), while the correct AL value was estimated based on the use of optical biometry data obtained by CASIA2. The postoperative IOL position (i.e., the depth of the anterior IOL surface position from the posterior surface of the cornea) was predicted by the newly developed calculation formula, including the preoperative ATA depth, PSD, and the correct AL and ACR values. The prediction was associated with an R^2^ value of 0.804 when the new formula was applied to the training set, and its accuracy was equivalent to or better than the value calculated by the proposed formula of the postoperative ACD developed by Goto *et al*. (R^2^ = 0.77)^19^. Additionally, the ICC between the predicted and true lens position yielded a high value, which indicates a good agreement. In the validation set, postoperative IOL position data were not collected because only preoperative biometric and postoperative refractive error data were collected. Thus, the predictive accuracy pertaining to the validation set was not estimated in this study, but it is expected to be smaller than the training set. The image (shape) obtained by CASIA2 was corrected based on a fixed refractive index at each segment. According to previous reports, the values obtained by CASIA2, including the CT, aqueous depth (AQD), and LT, exhibited good repeatability^21^. Furthermore, there were no statistically significant differences with those obtained with other instruments, such as ultrasonography and the Scheimpflug camera^21^. However, the measured values of the anterior and the posterior curvature radii of the crystalline lens and ESD generated concerns. The ESD is directly affected by the measurement error of the partially measured anterior and posterior curvature radii of the crystalline lens. This is because the equatorial surface of the crystalline lens is determined by drawing a line along the intersection point of the anterior and posterior curvatures. Thus, the predicted equatorial surface is distinct from the actual equatorial surface of the crystalline lens in reference to its anatomical location. In this study, ESD may not be incorporated in the regression formula for the prediction of the IOL position owing to the aforementioned reason^18^. Enriquez *et al*.^20^ reported that the preoperative anterior segment parameter (i.e., ACD, LT, lens equatorial plane position, and lens volume) were exceptionally valuable for estimating the IOL position based on three-dimensional OCT with full-shape image estimation. This suggests that the lens position depends on various anatomical characteristics. When the true crystalline lens parameters were obtained with the image estimation techniques applied on the entire lens shape, more accurate lens position estimates were obtained. The physical distances in each segment between the anterior corneal surface and the retinal photoreceptor layer were obtained by the combination of the corneal thickness measured by CASIA2 and the IOL thickness provided by the manufacturers, in addition to the corrected AL and IOL positions. It was considered that the new formula with paraxial ray tracing in the present study led to positive outcomes because the derived distances of each segment were accurate. Notably, the AL correction and IOL position estimation contribute largely to the accuracy of these outcomes. If the estimation of more accurate lens positions is possible, as discussed previously, this may lead to more accurate predictions. In addition, this study used paraxial ray tracing, but exact ray tracing—including the shapes, such as the spherical and aspheric shapes of the cornea and IOL—may yield more accurate predictions^15^. However, more data should be obtained and processed with special software that are more complex than paraxial ray tracing.

The predicted error of the refraction in the present study was calculated based on the difference of the actual objective refraction values after surgery and the expected refraction values before surgery. The conducted refraction measurements, which were based on subjective examinations with the use of the visual acuity chart, were not performed at a sufficiently long distance for the measurement of refraction at an infinite-distance focus. This is because the measured distance to the visual acuity chart is typically equal to 5.0 or 6.0 m. Hence, the measured refraction value based on subjectivity (manifested refraction) may overestimate hyperopia or underestimate myopia compared to the objective refraction value. However, this can be addressed by shifting the manifested refractive value or expected postoperative refractive value depending on the measurement distance. Besides, the best visual acuity during the measurements was set to be in the range of −0.3 to 0.0 based on the minimum angular resolution (logMAR) scale used in many clinics, including that used in our center (in our center it is commonly set to a value of −0.3 based on the logMAR scale). This may prevent the measurement of the refraction values at the best focus at infinite distances. This could be considered as another factor for the justification of the overestimation or underestimation of the measured refraction values. Additionally, the objective refraction values have an advantage in that they were measured in 0.01 D steps, whereas the manifested refraction values were measured in 0.25 D steps. Therefore, the manifested refraction value is not necessarily the same as the refraction value for an infinite-distance focus. The existing IOL calculation formulas can adjust the difference between the objective and the manifested refraction values using the lens constant. Accordingly, the expected postoperative refraction value calculated by the proposed formula is often compared to the manifested refraction value. However, the new formula proposed in the present study is a theoretical formula based on ray tracing and the use of the physical distances, while the lens constant was not used. We found that the expected postoperative refractive value calculated by this formula is in a good agreement with the objective refraction value. However, the manifested refraction had been established as a subjective evaluation test based on the consideration of the depth of focus related to the pupil size and higher-order aberrations. In future studies, this formula should be optimized to allow comparisons with the manifested refraction. We expect that the optimization may be able to address this issue by converting the objective refraction to subjective refraction.

No formula has yet been reported for calculating the IOL that is able to adapt to any eye type^15,22–27^. As previously mentioned, ELP was estimated with the SRK/T formula based on the Pythagorean theorem with K data, and was calculated to be deeper in steep K and shallower in flat K^9^. Thus, the prediction error for the SRK/T formula has the following characteristics: steep K values induce myopic errors, and flat K values induce hyperopic errors. In the present study, we have demonstrated the characteristics of the prediction error for the SRK/T formula. By contrast, the Barrett Universal II and Haigis formulas used the anterior segment parameters, such as the ACD and LT, to predict the ELP. Thus, the prediction errors for these formulas are not correlated with the K values. However, the prediction errors in the cases of the Barrett Universal II and Haigis formulas depended on AL. The Barrett Universal II formula may cause myopic prediction errors in long AL, and hyperopic prediction errors in short AL. Additionally, the Haigis formula may cause hyperopic prediction errors in long AL, and myopic prediction error in short AL. Note, however, that the error of the Haigis formula may have been overevaluated relative to those of other formulas. This is because the Haigis formula was not optimized with the triple constant set (a0, a1, and a2), based on the standard mode. Comparison with the previous reports of Melles *et al*.^26^ showed that the prediction error of the Haigis and Barrett Universal II formulas did not exhibit the same tendencies observed in the present study. The predicted errors were calculated based on the manifested refraction values in previous reports instead of the objective refraction values in the present study. This may have caused the differences with respect to previous reports. By contrast, the new formula may be used for any eye type because the prediction errors were not dependent on the AL and K values. Furthermore, the new formula is theoretically applicable as an IOL power calculation formula for other IOL types. In addition, it is applicable for eyes that have undergone refractive surgery, such as laser *in situ* keratomileusis (LASIK), or photorefractive keratectomy (PRK). With other IOL types or in post-LASIK cases, the correction of AL and the method of ray tracing required no changes. However, we may have to optimize the formula for the prediction of postoperative IOL positions because the IOL position largely depends on the mechanical design of the IOL platform, besides anatomical factors^28^; in the existing IOL calculation formula, this factor is normally accounted for by the lens constant, such as the A constant provided by the manufacturer. In post-LASIK cases, ACR can be excluded from the variables of the regression formula for the prediction of the IOL position. Accordingly, calculations can be conducted without the use of the obtained or predicted K values before surgery. Future studies will entail the validation of the prediction accuracy in the cases of implanted eyes with other IOL types, and applications to post refractive surgery eye cases. These studies will confirm the usability of the new formula.

The present study has some limitations. The population was Japanese and the AL was biased toward longer eyes^29^. The racial differences in the eye parameters may affect the estimation of postoperative ACD^30^. In addition, astigmatism affects optical performance, but this was not considered in this study, like most other ray tracing formulas. Future studies must focus on short eyes, racial diversity, and astigmatism, to improve the accuracy in this formula. The silicone three-piece AQ110NV IOL used in this study was also reported to induce postoperative myopic shifts and ACD changes^31^. In this study, the postoperative follow-up period was one month, and the prediction error may be overevaluated or underevaluated at this specific follow-up time. Lastly, the physical information on the optic design of IOL provided by the manufacturers is necessary for the IOL calculation method based on paraxial ray tracing in present study. This type of information is not easy to access, which may impede the universal use of this method. In other words, if this information is missing, we cannot use the method in its entirety.

In conclusion, this study demonstrated that paraxial ray tracing, including the IOL position predicted by AS–OCT, AL correction, and physical IOL information yielded excellent predictions of postoperative refraction on one IOL type. This IOL power calculation method does not need special software, and has the potential to reduce refractive errors after cataract surgery compared to existing IOL calculation formulas.

## Methods

The study was designed as a retrospective consecutive case series. It was approved by the Institutional Review Board of Sanno Hospital (approval number 17-S-6) and conformed to the guidelines of the Declaration of Helsinki. Informed consent was obtained from all participating patients. All patients were recruited from the Sanno Hospital after they had undergone uneventful cataract surgeries between April 2016 and March 2018. Participants were excluded from the study if they had a history of refractive surgery, intraocular surgery, or ocular pathology, and postoperative visual acuity worse than 20/40.

All patients underwent routine preoperative and postoperative ophthalmic examinations (at one month after surgery), including an objective refraction value measurement, corrected distance visual acuity measurements using a Landolt C chart, slit lamp examination, keratometry, intraocular pressure measurement, and funduscopy. The AL and ACR were measured with the use of a swept-source OCT device (IOLMaster 700, Carl Zeiss Meditec AG, Jena, Germany). ACR outcomes are indicated as K values in diopter units by the device software, based on the assumption of a keratometric index of 1.3375. The objective refraction value (in 0.01 D steps) was measured with the use of an autorefractor (TONOREF, Nidek Co., Ltd., Aichi, Japan). All three surgeons engaged in the study used a 2.8 mm postlimbal incision, a 5.0 mm centered curvilinear capsulorrhexis, and standard phacoemulsification with an in-the-bag IOL. The implanted IOL was a monofocal AQ110NV lens (STAAR Surgical, Monrovia, California, USA) which consisted of three pieces.

Clinical data were divided randomly into two groups, i.e., a training set and a validation set. The training set was used to develop the new IOL calculation formula and to optimize the constant values of the existing formula pertaining to the lens. The necessary data for the new IOL calculation formula were collected from the validation set and were used to validate the accuracy of the predictions. The SRK/T formula^9^ epitomized the third generation intraocular lens power (IOP) calculation formula, while the Haigis formula^32^ with an ELP, which was estimated without the K value, epitomized the fourth generation formulas. Both were open-sourced formulas. The Barrett Universal II formula^33,34^ has been a focus of attention as the most accurate formula. This is a closed-source formula, but can be calculated on the Asia Pacific Association of Cataract and Refractive Surgeons (APACRS) website. In this study, the predictive accuracy of a new IOL calculation formula was compared with the Barrett Universal II, Haigis, and SRK/T formulas.

### AS–OCT imaging and analysis

**Figure 5:**
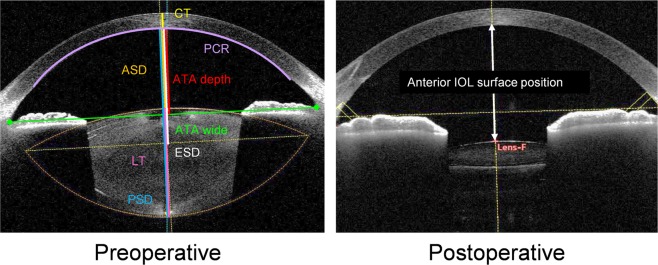
Parameters measured by the CASIA2 (CT: corneal thickness; PCR: corneal posterior curvature radius; LT: crystalline lens thickness; ATA width: angle-to-angle width; ATA depth: angle-to-angle depth; ASD: anterior surface depth; ESD: equatorial surface depth; PSD: posterior surface depth).

We imaged anterior segments with CASIA2 (TOMEY Corporation, Nagoya, Japan) preoperatively and at one month after surgery. The CASIA2 device, an AS–OCT device with a swept-source laser wavelength of 1310 nm, can measure depths as large as the anterior segment length, and is useful for the evaluation of the crystalline lens shape and actual postoperative IOL position. The CASIA2 software loads the image (shape) and corrects it based on a fixed refractive index at each segment. For preoperative anterior segment data, the corneal ACR, the PCR, CT, and LT were determined. The ATA depth was determined as the perpendicular distance between the posterior corneal surface and the intersection point of a line that joined the angle recesses on the cross-sectional horizontal image and the corneal vertex. The ASD for the crystalline lens was automatically calculated as the distance between the anterior surface of the lens and the posterior surface of the cornea. In addition, the PSD for the crystalline lens was calculated by adding the automatically calculated LT value to the ASD. The equatorial surface for the crystalline lens was determined by drawing a line through the intersection point of the anterior and the posterior curvature radii of the crystalline lens (i.e., the predicted equatorial surface). Additionally, the distance between the equatorial and the posterior surfaces of the cornea were quantitatively determined as the ESD of the crystalline lens. The anterior and posterior curvature radii of the crystalline lens were automatically traced to fit a circle with CASIA2 and were subsequently quantified (Fig. 5).

For the postoperative AS data, the anteriorly implanted IOL surface position was calculated as its distance from the posterior corneal surface (Fig. 5). If the crystalline lens axis was not identical to the optical axis, a straight-line distance was determined with the use of the crystalline lens axis.

### Development of IOL power calculation based on paraxial ray tracing

Assuming paraxial imagery, the refractive effect of any spherical surface can be calculated by the following formula^35^:

8 $$F=\frac{{n}_{2}-{n}_{1}}{r}$$

where *F* = refractive power of the surface in diopters (D), *n*_1_ = index of refraction of the material before the surface, *n*_2_ = index of refraction of the material after the surface, and *r* = radius of curvature in meters. Signage conventions dictate that the curvature radii of convex and concave surfaces are assigned to positive and negative values, respectively.

Vergence is another important concept in paraxial ray tracing, and it can explain ray transfer between the surfaces. It is described as the reciprocal of the “reduced” distance to the focal point, and is defined as^35^,

9 $$V=\frac{n}{d}$$

where *V* = vergence of paraxial rays in diopters, *d* = distance in meters from the vergence plane to the focal point, and *n* = refractive index of the material. When a refractive system (i.e., a lens) of power *F* is added to a bundle of rays of vergence *V*_1_, the vergence *V*_2_ of the rays exiting the lens can be calculated as the sum^35^,

10 $${V}_{2}={V}_{1}+F$$

Paraxial ray tracing was calculated using the above formulas based on the assumption that the retinal surface was a point light source. It was calculated using formulas applicable for a thick lens, whereby the cornea and lens were considered to have finite thicknesses with separate surface curvatures. The refractive surfaces are the posterior surfaces of the IOL, anterior surfaces of the IOL, and the posterior and anterior corneal surfaces. The refractive indices for the air, cornea, aqueous humor, vitreous humor, and IOL are equal to 1.0003, 1.376, 1.336, 1.336, and 1.413, respectively. An inverted value of the sign of vergence of the VD point (12 mm in front of the anterior corneal surface) is indicative of the expected postoperative refractive values. Measurement and analysis methods based on the use of these parameters are described below.

#### Corneal anterior and posterior refractive powers

The ACR was obtained by the ILM700. The device used a distance-independent telecentric keratometry system. The PCR was obtained by CASIA2, which can precisely delineate the boundaries of the cornea. In this study, the ACR obtained by the ILM700 was employed because the prediction error in the training set was lower than that using the ACR obtained by the CASIA2.

#### Axial length

The ILM700 determined OPL of AL and converted them into geometric/anatomical lengths based on assumed estimated values for the eye’s internal refractive indices. A unique average index (1.3549) was used based on the average group refractive index of the Gullstrand’s 24-mm model eye for an envelope of waves given the instrument’s infrared radiation wavelength of 780 nm. Additionally, the AL values measured with the ILM700 were approximated based on a regression equation that was formulated from precise, segmental, immersion, ultrasonic biometric measurements^32^. Therefore, the AL output from ILM700 (AL_IOLM_) did not always correspond to the true AL^32,36,37^.

We predicted the true AL based on reference to the report by Haigis *et al*.^32^ using the following procedure. The OPL of AL (AL_OPL_) can be calculated from AL_IOLM_:

11 $$A{L}_{OPL}=(A{L}_{IOLM}\times 0.9571+1.3033)\times 1.3549$$

The OPL of CT, ASD, and LT are calculated based on the multiplication of each value obtained by CASIA2 by the refractive index of each segment at the wavelength of 780 nm:

12 $$OPL=n\times L$$

where *n* = refractive index at 780 nm (CT = 1.3856, ASD = 1.3459, LT = 1.4070, vitreous thickness (VT) = 1.3445), and *L* = each length obtained by CASIA2. The length obtained by CASIA2 was corrected using the refractive index of each segment. Thus, it was defined as the true length in this study. The OPL of the vitreous length was calculated based on the subtraction of the OPL of CT, ASD, and LT from AL_OPL_. The vitreous length was calculated based on the division of the OPL of the vitreous length by the refractive index (1.3445). The correct AL was calculated based on the addition of the values of CT, ASD, and LT, as obtained by CASIA2, to the vitreous length, and based on the subtraction of the photoreceptor layer thickness (66 μm)^38^. Therefore, the correct AL value was calculated as follows,

13 $$V{T}_{OPL}=A{L}_{OPL}-1.3856\times CT-1.3459\times ASD-1.4070\times LT$$

14 $$Predictive\,true\,AL=CT+ASD+LT+\frac{V{T}_{OPL}}{1.3445}-0.066$$

#### Prediction for postoperative IOL position using anterior segment parameters

Based on the training dataset, the regression formula for the prediction of the IOL position was developed based on the results of the stepwise regression analysis (forward selection method as inclusion/exclusion criteria of P < 0.10) with the following nine parameters: PCR, CT, ATA depth, ATA width, ASD, ESD, and PSD, as measured by CASIA2; ACR measured by the ILM700, and the correct AL value.

#### IOL refractive power

Physical information on the optic design of IOL was provided by STAAR Surgical. This included the refractive index, thickness, and curvature of the anterior and posterior surfaces of the IOL for the given spherical power range.

### Optimization

In the existing formula, biometric data obtained by the ILM700 were used (AL, K, ACD, and LT in the Barrett Universal II formula; AL, K, and ACD in the Haigis formula; and AL and K in the SRK/T formula). Based on the training dataset, the optimized lens constants (a0 of the Haigis formula and A constants of the SRK/T formula) were determined such that the arithmetic prediction error was zero on average^39^. The Haigis formula was used in a standard mode with standard values for a1 (0.4) and a2 (0.1). The constant value of the Barrett Universal II formula (Lens factor) was determined by converting the optimized A constant of the SRK/T formula using the calculation tool of the Barrett Universal II formula available at the APACRS website.

### Statistical analyses

The Student’s t-test and the prop test were used to determine the significant differences between the training and the validation sets. The Pearson correlation coefficient (r) was used to determine the strength of the linear association between the anterior IOL surface position and each preoperative parameter. Multiple linear regression was used to estimate R^2^. Agreement between the predicted IOL position and anteriorly implanted IOL surface position were evaluated based on the ICC. The PE was calculated based on the subtraction of the predicted postoperative refractive value with the use of the actual IOL power implanted and the spherical equivalent of the actual postoperative refraction. The Med AE associated with the new formula was compared with those of the Barrett Universal II, Haigis, and SRK/T formulas based on the Wilcoxon signed-rank test. The percentages errors of the new formula (within ±0.50 D and ±1.00 D) were compared with those obtained by other formulas based on the McNemar test. The P value was corrected using the Bonferroni correction. A P value <0.05 was considered statistically significant. Statistical analyses were performed with a commercially available statistical software package (SPSS, version 24.0, IBM Corporation, Armonk, NY, USA).

## Supplementary information

Dataset 1.

## Data Availability

All data generated or analyzed during this study are included in this published article and its Supplementary Information files.
